# Brain activity and medical diagnosis: an EEG study

**DOI:** 10.1186/1471-2202-14-109

**Published:** 2013-10-01

**Authors:** Laila Massad Ribas, Fábio Theoto Rocha, Neli Regina Siqueira Ortega, Armando Freitas da Rocha, Eduardo Massad

**Affiliations:** 1 School of Medicine, University of São Paulo and LIM 01-HCMFMUSP, Dr. Arnaldo 455, 01246-903, São Paulo, Brazil; 2 RANI – Research on Artificial and Natural Intelligence, Jundiaí, Brazil

**Keywords:** Medical diagnosis, EEG analysis, Brain mapping, Human cognition, Decision-making

## Abstract

**Background:**

Despite new brain imaging techniques that have improved the study of the underlying processes of human decision-making, to the best of our knowledge, there have been very few studies that have attempted to investigate brain activity during medical diagnostic processing. We investigated brain electroencephalography (EEG) activity associated with diagnostic decision-making in the realm of veterinary medicine using X-rays as a fundamental auxiliary test. EEG signals were analysed using Principal Components (PCA) and Logistic Regression Analysis

**Results:**

The principal component analysis revealed three patterns that accounted for 85% of the total variance in the EEG activity recorded while veterinary doctors read a clinical history, examined an X-ray image pertinent to a medical case, and selected among alternative diagnostic hypotheses. Two of these patterns are proposed to be associated with visual processing and the executive control of the task. The other two patterns are proposed to be related to the reasoning process that occurs during diagnostic decision-making.

**Conclusions:**

PCA analysis was successful in disclosing the different patterns of brain activity associated with hypothesis triggering and handling (pattern P_1_); identification uncertainty and prevalence assessment (pattern P_3_), and hypothesis plausibility calculation (pattern P_2_); Logistic regression analysis was successful in disclosing the brain activity associated with clinical reasoning success, and together with regression analysis showed that clinical practice reorganizes the neural circuits supporting clinical reasoning.

## Background

The understanding of medical reasoning has been one of the greatest challenges of medical science, and the investigation of the neural systems responsible for this reasoning is one of the outmost challenges of neurosciences [1]. The purpose of this study was to combine theoretical knowledge about medical reasoning provided by Knowledge-Based (KBS) and Intelligent Computing Systems (ICS) and EEG Brain Mapping techniques available from neuroscience research to investigate how doctors manage clinical and radiological data during veterinary diagnosis.

KBSs consist of rule-based reasoning, case-based reasoning and model-based reasoning, while ICSs include genetic algorithms, artificial neural networks, fuzzy logic, Bayesian networks, among other components. Different combinations of the above methods have been used by previous studies to model and explain medical reasoning [1-4].

A study of diagnostic processing must allow free time for physicians to process the data at each stage of the diagnostic decision-making. In addition, allowing for hypothesis re-evaluation precludes the need for a rigid sequential protocol. These requirements make EEG a preferable tool for the recording and analysis of brain activity compared to fMRI investigation [5].

### Formalizing medical reasoning

Studies on medical diagnosis, independent of the chosen modeling structure, have shown that physicians usually obtain some key information (referred here as 'triggers’) from the history of the patient’s illness and physical examination, which leads to some hypotheses that can guide the search for additional data through laboratory tests, X-ray examinations, computerized tomography, etc [4,6-9]. Doctors gain valuable information from these auxiliary tests and compare this information with their initial hypotheses. Depending on the test results, the hypotheses are confirmed, discarded or re-evaluated [4,10]. In this approach, medical diagnosis is a characterization task that depends on the ability to identify the critical features (signs and symptoms) of a given case and the ability to appropriately weigh and combine these features to arrive at the correct diagnosis [4,6,8,9,11].

**Figure 1:**
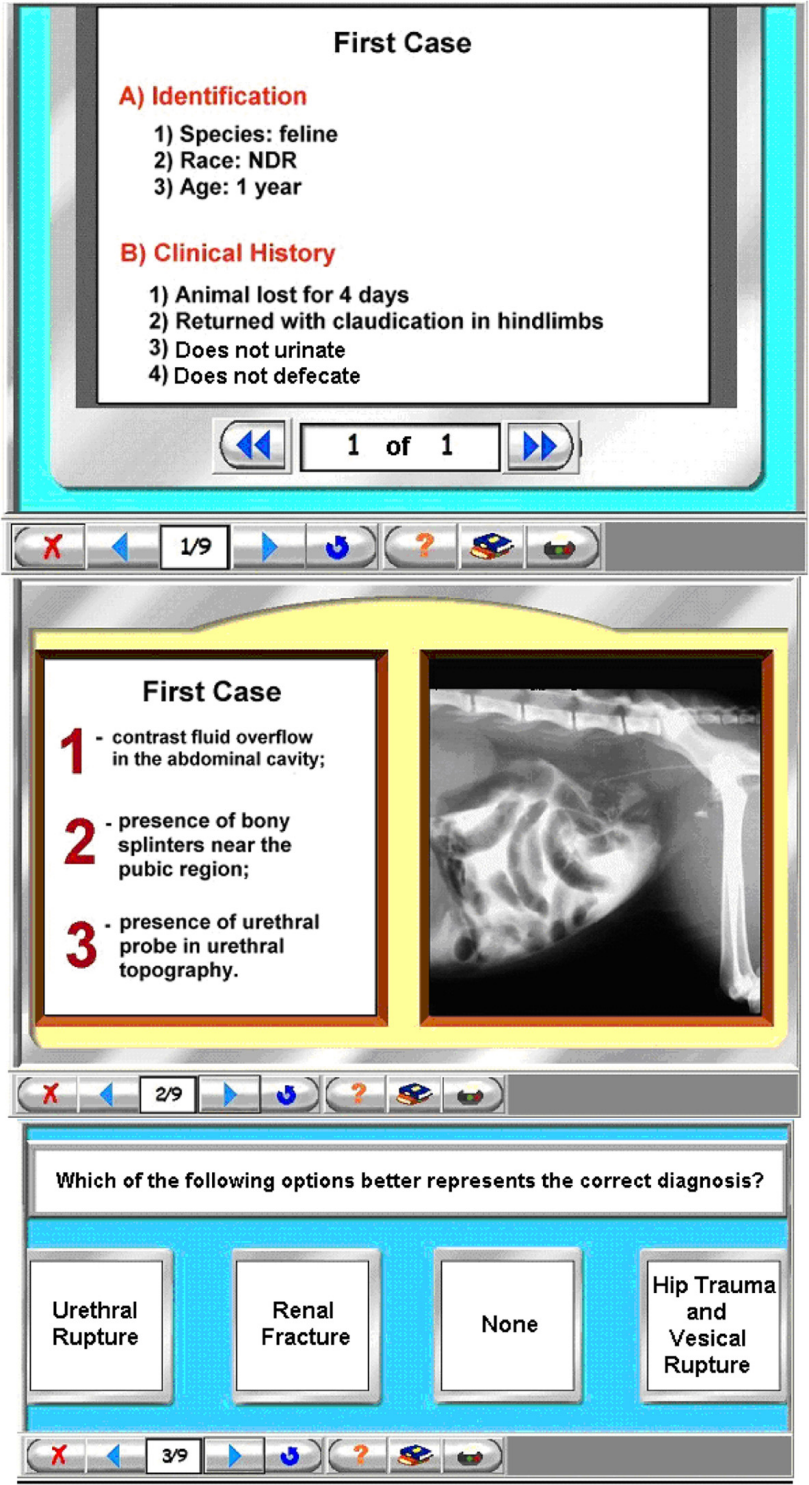
**A three-step experimental protocol for examining diagnostic decision-making.** The protocol includes reading a clinical history, analyzing an X-ray and determining a final diagnosis (see the main text for further explanation).

For example, in Figure 1, previous data including the *claudicating of the hind limbs* as well as a *absence of defecation* and *absence of urination* is thought to trigger a diagnosis of *hip trauma* with *urinary tract lesion*. The elucidation of these intermediate hypotheses requires an abdominal X-ray. When requesting the X-ray, the physician is aiming to detect potential *bone fractures* or *splinters* to confirm the *hip trauma* and the *contrast fluid overflow* in the abdominal cavity that indicates a *urinary tract lesion.*

**Figure 2:**
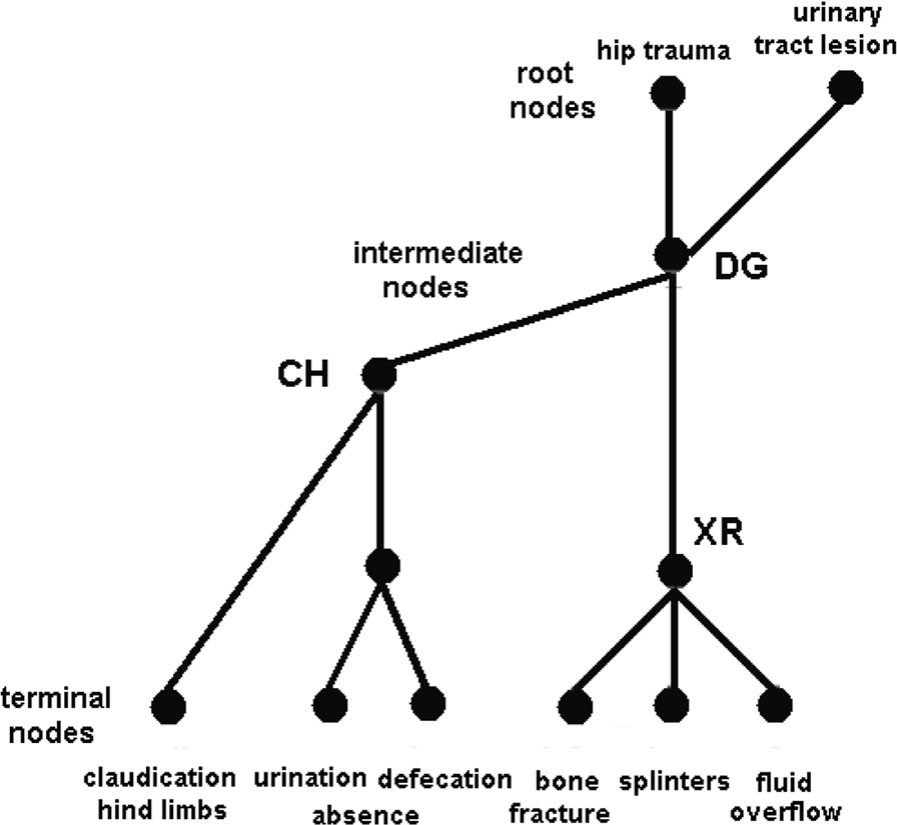
**An example of a reasoning graph RG.** Clinical data are combined to support the decision to ask for an X-Ray exam to obtain additional information to reach a final diagnostic) (see the main text for further explanation). CH means reading a clinical history, XR analyzing an X-ray and DG determining a final diagnosis.

According to Leão and Rocha [7] and Rocha [4], diagnostic reasoning involves a set of structured rules that involves networks of concepts and relationships that may be formalized by the reasoning graph (RG) shown in Figure 2. The trigger *claudicating of the hind limbs* prompts the doctor to explore the hypothesis of *hip trauma*, which is associated with positive information about *lack of defecation* and/or *lack of urination***,** and raises the supposition about the occurrence of the *urinary tract lesion*. The identification of *bone fractures* or *splinters* in the X-Ray confirms the *hip trauma* and the identification of *contrast fluid overflow* adds *urinary tract lesion* to the final diagnostic.

Reasoning of a clinical case is to navigate the RG and take into consideration at least two types of uncertainty:

a) uncertainty in the *identification* of the positive symptoms, clinical signs and auxiliary tests results; and

b) uncertainty in the *prevalence* of the positive symptoms, clinical signs and auxiliary tests results.

The lower the uncertainties in the identification and prevalence, the higher the certainty in the medical decision-making. For example, the lower the uncertainties in the identification and prevalence of *claudicating of the hind limbs, absence of defecation* and/or *absence of urination*, the higher the certainty of asking for an X-ray exam. In a similar manner, the lower the uncertainties in the identification and prevalence of the X-ray indication of *bone fractures* or *splinters*, the higher the certainty in *hip trauma* and/or *urinary tract lesion*.

Consistent with this line of reasoning, the clinical knowledge encoded by RG reads as a set of rules of the type:

*If (uncertainties in the identification and prevalence of claudicating of the hind limbs, absence of defecation and/or of urination) smaller than a*}, *then ask XR otherwise consider other diagnostic hypothesis*

*If { uncertainties in the identification and prevalence of claudicating of the hind limbs, lack of defecation and/or lack of urination bone fractures* or *splinters greater than a*}, *then revise diagnostic hypothesis otherwise consider hip trauma hypothesis and verify contrast fluid overflow*

$$\left\{\;u\left(\mathrm{HT}\right)=p\left(\mathrm{XR}\right)*\max\left[\;u\left(\mathrm{bs}\right)*p\left(\mathrm{bs}\right)\;\mathrm{or}\;u\left(\mathrm{bf}\right)*p\left(\mathrm{bf}\right)\right]\right\},$$

where u(HT), u(bs), u(bf) represent the uncertainties of hip trauma, bone splinters and bone fractures, respectively and p(HT), p(bs), p(bf) represent the probabilities of hip trauma, bone splinters and bone fractures, respectively,

otherwise

*If { uncertainties in the identification and prevalence of contrast fluid overflow is smaller thana*}, *then consider urinary tract lesion too*

otherwise disregard UTL

At each reasoning step, the RG navigation proceeds if the calculated total uncertainty is smaller than a given threshold *a*, otherwise the actual diagnostic hypothesis has to be revised [4]. Therefore, navigation to the next node is allowed if the identification uncertainty is low and the prevalence is high.

The complexity of the reasoning graph is directly linked to the number of nodes and edges between them [4,7] and it inversely correlates with medical expertise, being greater in the case of novices than in the case of senior clinicians. In other words, experts, compared to novices, use less clinical information nodes (to build simple rules to support their decision-making). Expertise in clinical decision-making is, therefore, the ability to correctly identify the critical feature of a given case and the ability to correctly weigh and combine these features to support the decision-making [8]. This is consistent with Mandin et al. [9] who proposed that '*resolution of clinical cases is markedly enhanced when medical knowledge becomes “elaborated,” or linked into networks of concepts and relationships … and then “compiled” into a high order knowledge structure that links abridged intricate networks into a scheme of relationships and diagnoses’.* They also considered that '*experts generally work forward utilizing schemes specific to problems within their domains of expertise and seldom rely on general search strategies’.*

Although there may be other strategies for medical decision-making, we used the above sequential reasoning strategy to guide the investigation protocol for this study.

### Medical reasoning brain mapping

Despite the fact that new brain imaging techniques have improved our ability to study of the processes of human decision-making, there have been a lack of studies that have investigated brain activity during diagnostic processing, with the recent exception of the work by Melo et al [12]. In the present study of diagnostic processing, the EEG activity was recorded as physicians were provided with:

1) clinical history information (**CH**) to trigger hypotheses that requires

2) X-ray data (**XR**) analysis to support

3) diagnostic decision-making (**DG**).

The study of diagnostic decision-making requires the analysis of brain activity prior to the decision-making to understand the cognitive processes associated with medical reasoning. In contrast, most if not all of the experiments involving EEG and decision-making described in the literature, have focused on the analysis of brain activity following decision-making or stimulus presentation [13-26]. Physicians should be allowed free time for process data and making decisions about diagnostic hypotheses, therefore, it is preferable to look the EEG backwardly from the moment the decision was completed to have a better understanding of the process. This was what Rocha et al. [5] proposed when they studied vote decision-making using EEG brain mapping techniques [27-30]. With this approach, they were able to disclose different patterns of brain activity associated with different voting decisions. These results motivated us to use the same EEG mapping technique to investigate diagnostic decision-making.

Rocha et al. [5] used a new EEG brain mapping technique to study the voting intention declared by a sample of Brazilian electors one week before the referendum day. Their results showed that the vote decision-making engaged networks responsible for calculating the uncertainty of identifying benefits and identifying risk prevalence of the decision of prohibiting or allowing firearm commerce and that the topology of such networks was vote-sensitive (i.e., **YES/NO**). According to Rocha et al. [5], the adequacy of decision-making depends on the evaluation of the risks and benefits. Principal component analysis (PCA) of the EEG activity revealed the existence of three different patterns (*P*_*i*_) of brain activity, which explained 80% of the data covariance associated with the voting decision. Rocha et al. [5] proposed that the networks disclosed by the *P*_1_ and *P*_2_ patterns, which were similar for both types of voters, are those in charge of calculating the adequacy or intention of each voting decision. Specifically, Rocha et al. [5] proposed that the *P*_2_ pattern was associated with the executive network in charge of controlling vote intention calculation, whereas the *P*_1_ pattern was associated with the calculation itself. In contrast, the *P*_3_ pattern differed for **YES** and **NO** voters, and the authors proposed that the networks disclosed by the *P*_3_ pattern were responsible for the risk and benefit evaluations.

In this study, we recorded the EEG activities of veterinary radiologists making decisions about diagnostic hypotheses from real clinical cases that required complementary X-ray information. We hypothesize that:

1. The brain activity recorded during the phases **CH**, **XR** and **DG** should not be significantly different because the reasoning process during these phases involve both the identification and prevalence uncertainty assessment and the combination of the two, to support decision-making;

2. PCA should reveal distinct brain activity patterns associated with the uncertainty assessment and handling;

3. Regression analysis should complement the above hypotheses by disclosing a correlation between the result (right or wrong) of the diagnostic decision-making and the EEG activity recorded by each electrode during the **CH**, **XR** and **DG**.

4. This regression analysis should be sensitive to the clinical experience of the volunteers, as measured by the number of years of practice.

After this introduction, we will describe the methods we employed, followed by the results of our study as well as a discussion of our findings and their implications for diagnostic decision-making processes.

## Methods

Twenty-nine veterinary radiologists of both sexes (16 females and 13 males), with clinical practice (*expertise*) varying from 0.5 to 15 years, were invited to read fourteen clinical histories that required X-ray information to determine a diagnosis (Figure 1). The cases were selected by a senior radiologist and were submitted to a several other radiologists who determined that these cases were of adequate difficulty for the purposes of this study.

After reading each clinical history, the volunteers examined a radiographic image that had one or more radiographic features that were relevant for the diagnostic decision-making. The volunteers had to select a number that was associated with the feature they considered relevant and to drag it to a corresponding image in the X-ray. In the sequence, they were presented with four possible diagnostic hypotheses. Their options were to select one of the hypotheses as the correct diagnosis or to completely or partially review the case. Their EEGs (20 electrodes placed according to the 10/20 system; ear lobe reference; impedance smaller than 10 kOhm; low bandpass filter: 50 Hz; sampling rate of 256 Hz and 10 bits resolution) were recorded during the entire task and at the ending moments (that is, the moment of time participants move from one phase to the next) of the clinical history reading phase,*T*_*CH*_, the X-Ray inspection phase, *T*_*XR*_, and the decision phase, *T*_*DG*_, and at the selected diagnosis were also annotated. EEG was visually inspected and bad recorded epochs were discarded on an single basis. The selected diagnosis (**d**) was compared to the corrected diagnosis for each case and re-coded as **d = 1**, if the selection was correct or **d = 0**, if otherwise.

Signals from a multi-channel EEG are unavoidably correlated due to the fact that the recordings from each electrode are generated by local field potentials or source signals (*s*_*i*_) from several distinct cortical areas. The source signals *s*_*i*_ can be summed up and projected to the electrodes. This is due to the radial orientation of pyramidal cells relative to the cortical surface. Were not for this possibility, the local fields would partially or completely cancel each other out. In this context, EEG data *d*_*i*_(*t*) recorded at a single electrode *e*_*i*_ are a simple weighted sum of underlying (*k*) cortical source signals *s*_*i*_ that are active at time *t*, that is:

(1) $$d_{i}\left(t\right)=\sum_{i=1}^{k}w_{i}s_{i}\left(t\right)$$

where *w*_*i*_ stands for the weight assigned to source *s*_*i*_. The number *k*of active sources are determined by the task being currently processed by the brain.

A major question in EEG analysis is to locate EEG signal sources *s*_*i*_. In an attempt to answer this question, different techniques have being used to correlate these to specific electrical sources locations. Recent studies have shown that source *s*_*i*_ varies for each EEG components [31-35] and some of these studies have shown that different sources may be linked to the same component. Therefore, each electrode may record signals from sources that have different spatial and temporal distributions, and different electrodes may record signals from the same source. In this way EEG data *d*_*i*_(*t*) recorded by each single electrode *e*_*i*_ may provide different or redundant information about the sources activated by the task being currently processed. In this context, a key datum that may be obtained from the EEG about how the task is being processed is the amount of information *H*(*e*_*i*_) each electrode may provide about the sources *s*_*i*_[5,30].

To study the EEG correlates of cognition one has to investigate the relations between *k* EEG source components *s*_*i*_ supposed to be involved in the cognitive task solving and behavioral variables that provide information about how the cognitive task was solved. To be sure that all *k* activated *s*_*i*_ are indentified requires the use of all available EEG analytical tools to provide information about distinct sources *s*_*i*_. In addition, the statistical complexity of the investigation increases as the number of EEG and behavioral variables increases. Therefore, at least as an initial approach, it is interesting to avoid the identification problem and to reduce the number of studied EEG variables.

Because EEG data are assumed to be a weighted sum of the electrical activity of different sources, correlation analysis of the EEG activity *d*_*i*_(*t*) recorded by the different electrodes *e*_*i*_ may be used to calculate *H*(*e*_*i*_) in order to summarize information provided by each electrode *e*_*i*_ about all involved sources *s*_*i*_ into a single variable as proposed by Rocha et al [5]. The rationality is the following.

The Pearson correlation R is +1 in the case of a perfect positive (increasing) linear relationship (correlation), -1 in the case of a perfect decreasing (negative) linear relationship (anticorrelation),and some value between -1 and +1 in all other cases, indicating the degree of linear dependence between the variables. As it approaches zero there is less of a relationship (closer to uncorrelated). The closer the coefficient is to either -1 or +1, the stronger the correlation between the variables. In this context, the correlation strength *r* is equal to |*R*|. If data *d*_*i*_(*t*),*d*_*j*_(*t*) furnished by two electrodes *e*_*i*_, *e*_*j*_ provide equivalent information about sources *s*_*i*_ then Pearson correlation coefficient *R*_*i,j*_ calculated for *d*_*i*_(*t*),*d*_*j*_(*t*) will approach ±1, otherwise it will approach 0. The highest uncertainty about the information equivalence provided by *e*_*i*_, *e*_*j*_ occurs when the correlation strength *r*_*i,j*_ approaches 0.5. Therefore, in the same line of reasoning used by Shannon to define the amount of information provided by a random variable, it is proposed that the informational equivalence, *H*(*r*_*i,j*_) of *d*_*i*_(*t*),*d*_*j*_(*t*) furnished by *e*_*i*_, *e*_*j*_ is the expected value *E*(*I*(*r*_*i,j*_)) of the information *I*(*r*_*i,j*_) provided by *r*_*i,j*_. Therefore:

(2) $$\begin{array}{l} H\left(r_{i,j}\right)=E\left(I\left(r_{i,j}\right)\right)\\ \;=-\left[r_{i,j}\log_{2}\left(r_{i,j}\right)+\left(1-r_{i,j}\right)\log_{2}\left(1-r_{i,j}\right)\right] \end{array}$$

such that if *r*_*i,j*_ *=* 0.5 then *H*(*r*_*i,j*_) = 1 and if *r*_*i,j*_ *=* 1or *r*_*i,j*_ *=* 0 then *H*(*r*_*i,j*_) = 0.

Now, given

(3) $${\bar{r}}_{i}=\frac{\sum_{j=1}^{19}r_{i,j}}{19}$$

the entropy of ${\bar{r}}_{i}$ is

(4) $$H\left(\bar{r_{i}}\right)=-K\left[{\bar{r}}_{i}\log_{2}{\bar{r}}_{i}+\left(1-{\bar{r}}_{i}\right)\log_{2}\left(1-{\bar{r}}_{i}\right)\right]$$

where *K* is a constant. The entropy quantifies the mean informational equivalence from *d*_*i*_(*t*) concerning that provided by all other *d*_*j*_(*t*), because the different electrodes (information channels) provide different, but correlated, information about *s*_*i*_.

In this context, we propose that

(5) $$H\left(e_{i}\right)=\frac{\sum_{j=1}^{19}\left[H\left({\bar{r}}_{i}\right)-H(r_{i,j}\right)]}{19}$$

quantifies the information provided by *d*_*i*_(*t*) about the sources *s*_*i*_ involved in a cognitive task solving, because

a) if *r*_*i,j*_*= k* for all all *e*_*j*_ then ${\bar{r}}_{i}=k$, $H\left(r_{i,j}\right)=H\left(\bar{r_{i}}\right)$ for all *e*_*j*_, and consequently *H*(*e*_*i*_) = 0. This indicates that *d*_*i*_(*t*) *e*_*i*_ does not provide any additional information about the sources *s*_*i*_;

b) for all other conditions0 < *H*(*e*_*i*_) <1and quantifies the information provided by *d*_*i*_(*t*) about the sources *s*_*i*_.

While Event Related Activity and Spectral Band Analysis may provide information about specific and localized sources involved in a task solving, *H*(*e*_*i*_) provides information about the spatial and temporal distribution of these sources, therefore, provides information about how different sets of neuron enroll themselves in a widely distributed network to solve a task [30]. Another interesting *H*(*e*_*i*_) property is that it summarizes information about all sources into a single variable, simplifying many analysis (e.g., regression analysis, principal component analysis, etc.) involving behavioral and neural variables [5,30].

An EEG time epoch of two seconds before the marks *T*_*CH*_,*T*_*XR*_ and *T*_*DG*_ was selected for analysis. The normalized values of *H*(*e*_*i*_) were used to construct brain maps according to the procedures described by previous studies [5,27-30].

The mean entropy *H*_0_(*e*_*i*_) was computed from a *hypothetical brain* artificially constructed by randomly shuffling the EEG recorded activity across the participants. Next, the Z-scores between the observed mean entropy *H*(*e*_*i*_) were computed for each of the EEG epochs (**CH, XR** and **DG**) and *H*_0_(*e*_*i*_). This was done to estimate the role of chance in determining the values of the observed *H*(*e*_*i*_). The minimum Z-score for all of these calculations was 1.85 (p = 0.0322, one-tailed test). Therefore, it was concluded that *H*_0_(*e*_*i*_) differed significantly from *H*(*e*_*i*_) for all EEG epochs, rejecting the role of chance in determining the observed results.

Principal Component Analysis (PCA) is a statistical tool to investigate patterns of covariation in a large number of variables and to determine if information may be condensed into small sets of these variables called principal components. This transformation is defined in such a way that the first principal component is the one that accounts for as much of the variability in the data as possible), and each succeeding component in turn explain the subsequent amount of variance possible under the constraint that it be orthogonal to (i.e., uncorrelated with) the preceding components. Factorial mappings are proposed to represent the activity of the neural circuits enrolled in a cognitive task because they condensed the information provided by the electrodes sampling this neural activity. In this ways, factorial analysis does not map brain areas activated by a cognitive task, but provides information to disclose the activity of circuits composed by neurons distributed on different areas of the brain recruited by the cognitive task, because *H*(*e*_*i*_) measures the amount of information provided by *e*_*i*_ about spatial and temporal distribution of *s*_*i*_. PCA was applied here to study the co variation of *H*(*e*_*i*_) calculated for each of the 406 decision (29 subjects times 14 clinical cases) during each of the selected epochs **CH**, **XR** and **DG**. The factorial brain maps were constructed to describe the results of the PCA using the procedures employed by Rocha et al. [5,27-30], taking the loading values (the correlation coefficients between the electrodes (rows) and factors (columns)) *f*_*i*_(*e*_*i*_) of *H*(*e*_*i*_) for each of the factors F_j_(*j* = 1,2,3) into account. To estimate the potential similarities between each factorial brain mapping that was associated to each F_j_(*j* = 1,2,3,4) for each EEG time epoch, we used Pearson correlation coefficients, which were calculated for their respective loading values *f*_*i*_(*e*_*i*_). In addition to the loadings to summarize our variables (the electrode entropies *H*(*e*_*i*_)) we calculated the individual scores to summarize relationships among the 406 observations (different clinical decisions times individuals). The loading and score plotting were used to check the existence of differences between the three phases.

A logistic regression analysis was used to study the association between the diagnostic decision **d** (dependent variable; wrong = 0, right = 1) and *H*(*e*_*i*_), which was calculated for each of the electrodes (independent variables) and adjusted for the variable *expertise* (potential confounder)*.* Logistic regression analysis, therefore, may disclose the electrodes that provide information that is important in decision-making. The normalized values of the regression coefficients *β*_*i*_ multiplied by the entropy, *β*_*i*_*H*(*e*_*i*_) were used to generate the color-coded brain mapping images to display the results of this regression analysis. Those that were statistically positive *β*_*i*_*H*(*e*_*i*_) were color coded from green (normalized *β*_*i*_*H*(*e*_*i*_) tending to 0) to dark blue (normalized *β*_*i*_*H*(*e*_*i*_) tending to +1). Those that were statistically negative *β*_*i*_*H*(*e*_*i*_) ranged from pink (normalized *β*_*i*_*H*(*e*_*i*_) tending to 0) to dark red (normalized *β*_*i*_*H*(*e*_*i*_) tending to -1), and those that were statistically non-significant *β*_*i*_*H*(*e*_*i*_) are shown in orange. The Holm-Bonferroni method and the calculation of the relative risks (exp*β*_*i*_*H*(*e*_*i*_))) with their respective confidence intervals were used to calculate the significance of these statistical inferences.

This work was reviewed and approved by the Institutional Ethical Review Board. All of the volunteers signed a written informed consent.

## Results

The median reading time of the clinical history was 11 seconds and the median time spent on the X-ray analysis was 24 seconds. The mean time for choosing the correct diagnostic hypothesis was 8 seconds and the average performance for correctly diagnosing the cases used in this study was 71.5%. These short time periods in each phase corroborate the hypothesis that cases were of adequate difficulty for the purposes of this study because difficult tasks are usually time consuming. In addition, only 31% of the doctors had reviewed a clinical history and/or X-ray date once, which demonstrates that the decision-making required some cognitive effort. The need to review the cases also varied according to the number of years the doctors had been practicing.

**Figure 3:**
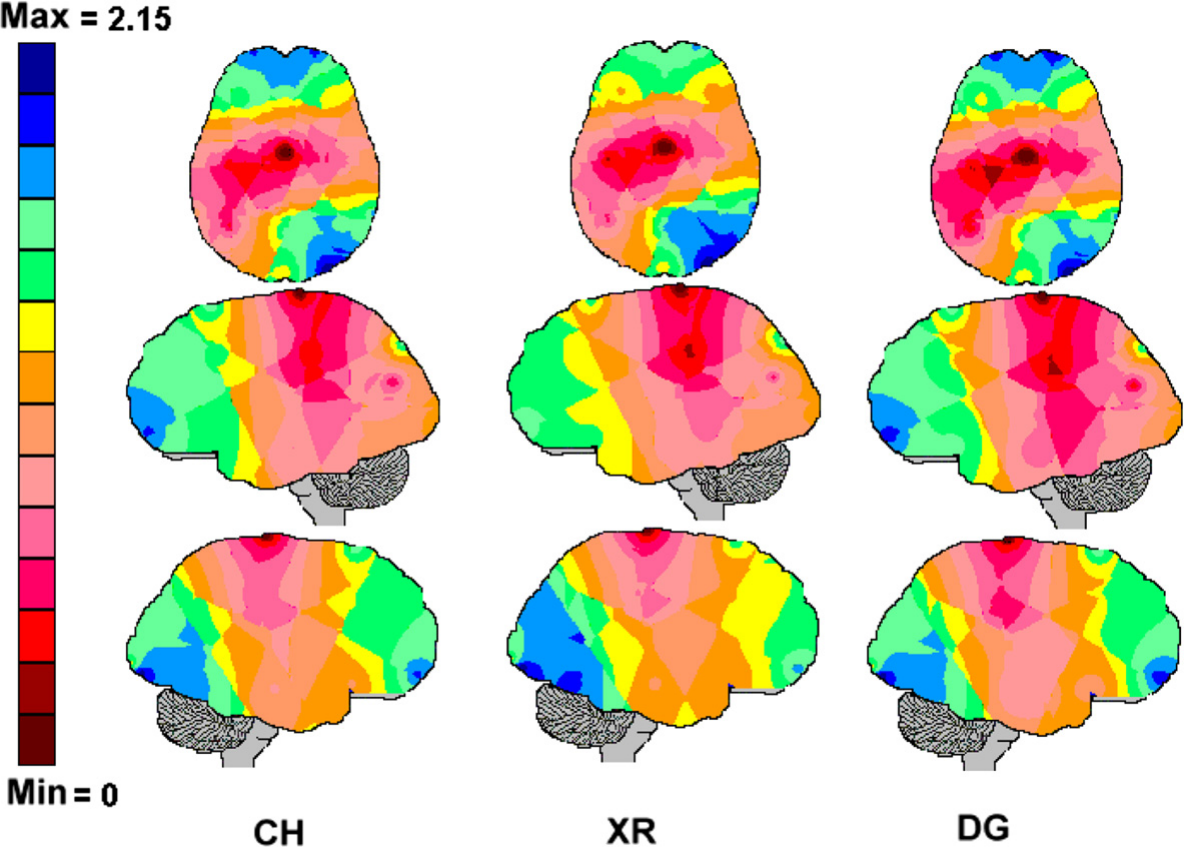
**The entropy brain maps associated with the clinical history reading (CH), X-ray analysis (XR) and diagnosis decision-making (DG).** The normalized entropy values obtained for each electrode were color coded such that the dark-blue areas were associated with the highest entropy values and the dark-red areas were associated with the lowest entropy values.

Table 1 shows the entropy values *H*(*e*_*i*_) calculated for each electrode *e*_*i*_ and each stage of the diagnostic decision-making process. The normalized $\bar{H}\left(e_{i}\right)$ values were obtained for each electrode and for each stage (CH, XR or DG) and were used to generate the entropy brain maps shown in Figure 3. The Pearson correlation coefficients calculated for the entropy values and the different stages (CH, XR and DG) are shown in Table 1.

**Table 1:** **Entropy *H*(*e***_***i***_**) (and normalized entropy**$\bar{H}\left(e_{i}\right)$**) results from the EEG activity associated with the clinical history reading (CH), X-ray analysis (XR) and diagnostic decision-making (DG) as well as the Pearson correlation coefficient (R) values between the different stages of the diagnostic decision-making process**

	***H*(*e***_***i***_**)**			$\bar{H}\left(e_{i}\right)$		
	**CH**	**XR**	**DG**	**CH**	**XR**	**DG**
C3	1.56	1.62	1.6	0.13	0.09	0.10
C4	1.63	1.75	1.65	0.26	0.31	0.20
CZ	1.49	1.57	1.55	0.00	0.00	0.00
F3	1.83	1.85	1.81	0.63	0.48	0.51
F4	1.8	1.85	1.82	0.57	0.48	0.53
F7	1.86	1.94	1.92	0.69	0.64	0.73
F8	1.73	1.83	1.75	0.44	0.45	0.39
FP1	1.95	1.99	1.99	0.85	0.72	0.86
FP2	1.96	2.03	2.01	0.87	0.79	0.90
FZ	1.91	1.96	1.94	0.78	0.67	0.76
O1	1.75	1.83	1.8	0.48	0.45	0.49
O2	2.03	2.15	2.06	1.00	1.00	1.00
OZ	1.78	1.88	1.82	0.54	0.53	0.53
P3	1.57	1.69	1.59	0.15	0.21	0.08
P4	1.87	2.00	1.9	0.70	0.74	0.69
PZ	1.89	2.02	1.91	0.74	0.78	0.71
T3	1.66	1.78	1.69	0.31	0.36	0.27
T4	1.70	1.82	1.71	0.39	0.43	0.31
T5	1.73	1.82	1.72	0.44	0.43	0.33
T6	1.92	2.06	1.95	0.80	0.84	0.78
				R		
			CH	XR	DG	
		CH	1.00	0.97	0.99	
		XR	0.97	1.00	0.96	
		DG	0.99	0.97	1.00	

The Pearson correlation coefficients were very high for all of the combinations, which allowed us to conclude that *H*(*e*_*i*_) did not substantially change during the different diagnostic decision stages. This trend implied that the associated EEG brain maps, shown in Figure 3, were similar to each other.

The brain maps in Figure 3 show that the *H*(*e*_*i*_) attained its highest values for two groups of electrodes: the frontal bilateral electrodes and right parietal-occipital electrodes. In the case of frontal bilateral electrodes, the highest *H*(*e*_*i*_) values were obtained for FP1 and FP2. In the case of the right temporal-parietal-occipital electrodes, the highest *H*(*e*_*i*_) value was obtained for O2. A third group of electrodes was identified when the lowest *H*(*e*_*i*_) values were considered. This group included the CZ, C3 and P3.

The PCA results of the EEG activity associated with the clinical history reading (CH), X-ray analysis (XR) and diagnostic decision-making (DG) are shown in Table 2. Table 2 also shows the Pearson correlation correlation coefficients between the PCA factors for CH-XR, CH-DG and XR-DG.

**Table 2:** PCA results of the EEG activity associated with clinical history reading (CH), X-ray analysis (XR) and diagnostic decision-making (DG)

		**CH**				**XR**				**DG**	
	**1**	**2**	**3**		**1**	**2**	**3**		**1**	**2**	**3**
C3	0.31	0.68	0.02	C3	0.23	0.80	0.05	C3	0.20	0.78	0.16
C4	0.06	0.71	0.30	C4	0.11	0.89	0.24	C4	0.08	0.84	0.28
CZ	0.13	0.75	0.33	CZ	0.14	0.85	0.27	CZ	0.10	0.83	0.31
F3	0.88	0.13	0.28	F3	0.84	0.27	0.29	F3	0.86	0.14	0.31
F4	0.89	0.18	0.18	F4	0.86	0.28	0.21	F4	0.87	0.22	0.22
F7	0.85	0.29	0.23	F7	0.87	0.28	0.18	F7	0.87	0.24	0.20
F8	0.73	0.37	0.17	F8	0.72	0.38	0.28	F8	0.77	0.35	0.13
FP1	0.82	0.05	0.48	FP1	0.85	0.05	0.46	FP1	0.80	0.05	0.52
FP2	0.81	0.09	0.48	FP2	0.85	-0.12	0.42	FP2	0.80	0.02	0.50
FZ	0.91	0.12	0.24	FZ	0.89	0.24	0.24	FZ	0.88	0.12	0.27
O1	0.51	0.25	0.78	O1	0.56	0.23	0.76	O1	0.49	0.25	0.80
O2	0.34	0.12	0.89	O2	0.38	0.25	0.85	O2	0.33	0.22	0.88
OZ	0.27	0.09	0.80	OZ	0.28	0.31	0.76	OZ	0.24	0.23	0.82
P3	0.24	0.67	0.59	P3	0.27	0.69	0.56	P3	0.25	0.66	0.61
P4	0.29	0.41	0.78	P4	0.30	0.49	0.75	P4	0.26	0.45	0.80
PZ	0.29	0.37	0.79	PZ	0.35	0.44	0.74	PZ	0.30	0.40	0.80
T3	0.20	0.83	0.18	T3	0.23	0.81	0.27	T3	0.23	0.85	0.20
T4	0.06	0.59	0.63	T4	0.13	0.79	0.44	T4	0.10	0.74	0.51
T5	0.34	0.72	0.43	T5	0.40	0.68	0.40	T5	0.38	0.64	0.51
T6	0.38	0.32	0.81	T6	0.48	0.36	0.73	T6	0.39	0.37	0.79
	P1	P2	P3		P1	P2	P3		P1	P2	P3
		% total	Cumul.			% total	Cumul.			% total	Cumul.
	Eigen	Varia	%		Eigen	Varia	%		Eigen	Varia	%
1	14.15	70.75	70.75	1	14.77	73.85	73.85	1	13.92	69.62	69.62
2	1.65	8.27	79.02	2	1.58	7.92	81.77	2	1.84	9.20	78.82
3	1.30	6.48	85.50	3	1.03	5.15	86.92	3	1.35	6.74	85.56
		CH-XR				CH-DG				XR-DG	
	1	2	3		1	2	3		1	2	3
	0.99	0.94	0.98		0.99	0.97	0.98		0.99	0.98	0.98

**Figure 4:**
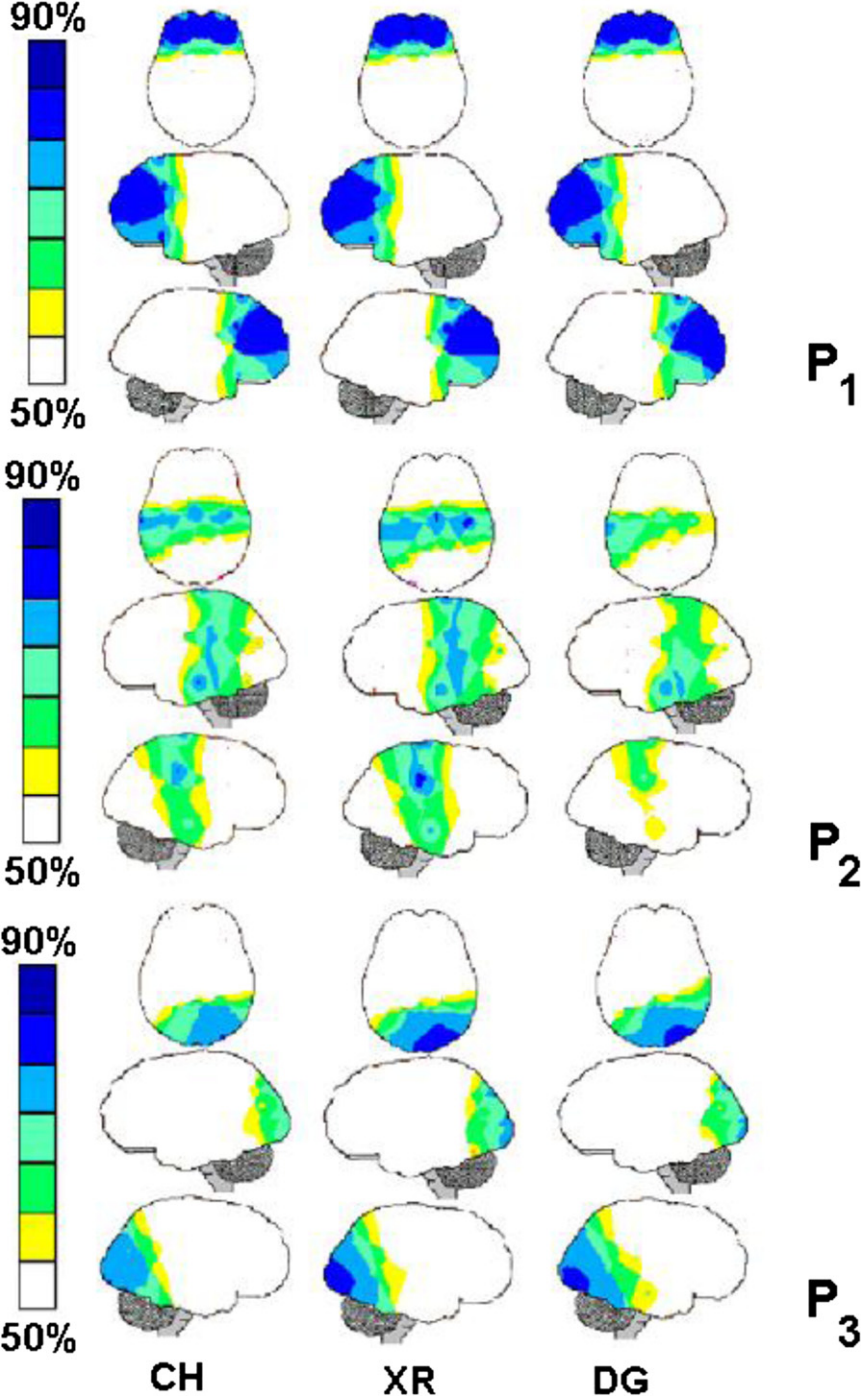
**PCA brain mappings (P**_**1**_**, P**_**2**_**, P**_**3**_**) associated with clinical history reading (CH), X-ray analysis (XR) and diagnostic decision-making (DG).** The normalized loadings for each PCA factor greater than 0.5 in Table 2 were color-coded from yellow to dark-blue to generate the brain maps. The dark-blue areas depicted the electrodes where entropy *H*(*e*_*i*_) was heavily loaded in the corresponding factor.

Three factors accounted for more than 85% of the total variance in the CH, XR and DG EEG entropy data. The eigenvalues varied from 14.77 for XR-P_1_ to 1.03 for XR-P_3_. Each of these factors characterized a distinct pattern of brain activity co variation and was used to generate the brain mappings (P_1_, P_2_, P_3_), as shown in Figure 4. The Pearson correlation coefficients in Table 2 clearly show that the PCA brain mappings were very similar when the CH, XR and DG were compared.

The P_1_ brain mappings show that *H*(*e*_*i*_) calculated for the frontal electrodes FP1, FP2, F3, F7, FZ, F4 and F8 covaried together; the P_2_ brain mappings showed a high *H*(*e*_*i*_) co variation for the electrodes T3, T4, T5, C3, C4 and CZ; and the P_3_ brain mappings showed a high *H*(*e*_*i*_) co variation for the electrodes O1, O2, OZ, P3, P4, and PZ.

**Figure 5:**
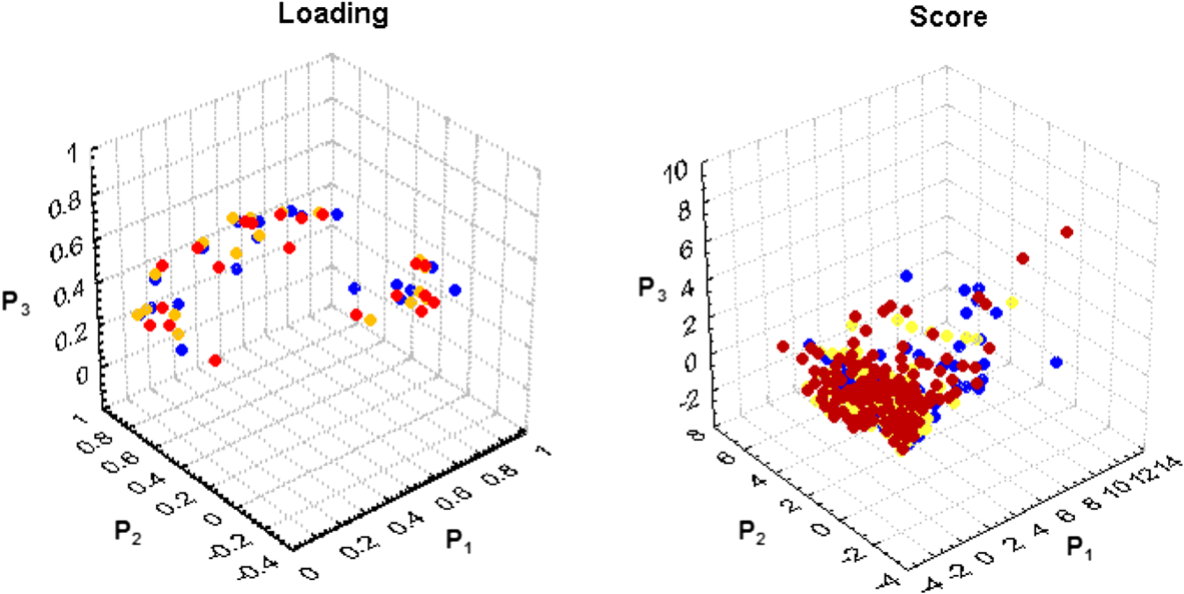
**Loading and Score 3-D plots for the 3 patterns resulting from PCA brain mappings (P**_**1**_**, P**_**2**_**, P**_**3**_**) associated with clinical history reading (CH) in blue, X-ray analysis (XR) in yellow and diagnostic (DG) in red.**

Figure 5 show the 3-D plots for loadings and scores, calculated for the 3 patterns P_i_ shown in Figure 4.

As can be seen in Figure 5, both the loading and score 3-D plots show cluster of variables and individuals from the 3 diagnostic decision phases that are overlapped. No difference between the 3 phases is remarkable with this technique.

Table 3 showed the results of the logistic regression analysis between the diagnostic decision, **d** (dependent variable; wrong = 0, right = 1), and *H*(*e*_*i*_) calculated for each of the electrodes (independent variables). The results of the logistic regression analysis between **d** and *H*(*e*_*i*_) was calculated for each of the electrodes and controlled for the variable *expertise* (potential confounder), as shown in Table 4*.* The expertise contributed to an increase R^2^ and to an increase of the value of **d**.

**Table 3:** **Logistic regression analysis between the diagnostic decision and *H*(*e***_***i***_**)**

**Dependent variable: d**				
**Final loss: 645.85**		**R = 0.37**	**R**^**2**^ **= 0.138**	
				**H-B**
	***β***_***i***_	**Std.Err.**	**p-level**	$\beta_{i}\bar{H}\left(e_{i}\right)$
Intercept	1.39	0.09	0.234	
C3	-1.97	0.39	0.000*	0.01
C4	2.41	0.29	0.000*	1.00
CZ	-0.65	0.32	0.040	0.50
F3	1.11	0.32	0.000*	0.71
F4	0.36	0.35	0.301	0.50
F7	0.15	0.25	0.535	0.50
F8	-0.17	0.25	0.497	0.50
FP1	-0.20	0.22	0.375	0.50
FP2	0.18	0.27	0.505	0.50
FZ	-0.71	0.29	0.016	0.50
O1	1.06	0.30	0.001*	0.69
O2	-2.02	0.29	0.000*	0.00
OZ	2.15	0.33	0.000*	0.94
P3	1.23	0.42	0.004*	0.73
P4	0.64	0.29	0.028	0.50
PZ	-1.76	0.38	0.000*	0.06
T3	0.36	0.28	0.190	0.50
T4	-0.91	0.32	0.004*	0.25
T5	-0.71	0.25	0.004*	0.30
T6	0.45	0.27	0.094	0.50

The values $\beta_{i}\bar{H}\left(e_{i}\right)$ are the normalized values of the product *β*_*i*_*H*(*e*_*i*_). The statistically significant inferences according to the Holm-Bonferroni (H-B) method are marked with asterisks.

**Table 4:** **The logistic regression analysis between the diagnostic decision and *H*(*e***_***i***_**) controlled for *expertise***

**Dependent variable: d**				
**Final loss: 595.43**		**R = 0.45**	**R**^**2**^ **= 0.205**	
				**H-B**
	***β***_***i***_	**Std.Err.**	**p-level**	$\beta_{i}\bar{H}\left(e_{i}\right)$
Intercept	0.05	0.13	0.677	
*Expertise*	0.24	0.02	0.000	
C3	-0.21	0.34	0.531	0.50
C4	1.43	0.22	0.000*	1.00
CZ	-0.88	0.29	0.003*	0.11
F3	0.75	0.30	0.008*	0.74
F4	0.07	0.29	0.815	0.50
F7	0.08	0.21	0.687	0.50
F8	0.34	0.19	0.009*	0.58
FP1	0.12	0.24	0.613	0.50
FP2	-0.07	0.26	0.801	0.50
FZ	-0.86	0.30	0.005	0.50
O1	0.33	0.27	0.231	0.50
O2	-0.65	0.26	0.013	0.50
OZ	1.11	0.30	0.000*	0.88
P3	-0.19	0.43	0.653	0.50
P4	0.06	0.29	0.825	0.50
PZ	-0.59	0.31	0.063	0.50
T3	0.06	0.25	0.823	0.50
T4	-1.17	0.33	0.000*	0.00
T5	-0.43	0.25	0.078	0.50
T6	1.27	0.32	0.000*	0.94

The values $\beta_{i}\bar{H}\left(e_{i}\right)$ are the normalized values of the product *β*_*i*_*H*(*e*_*i*_), which were estimated for those inferences according to the Holm-Bonferroni (H-B) method. The statistically significant inferences are marked with asterisks.

**Figure 6:**
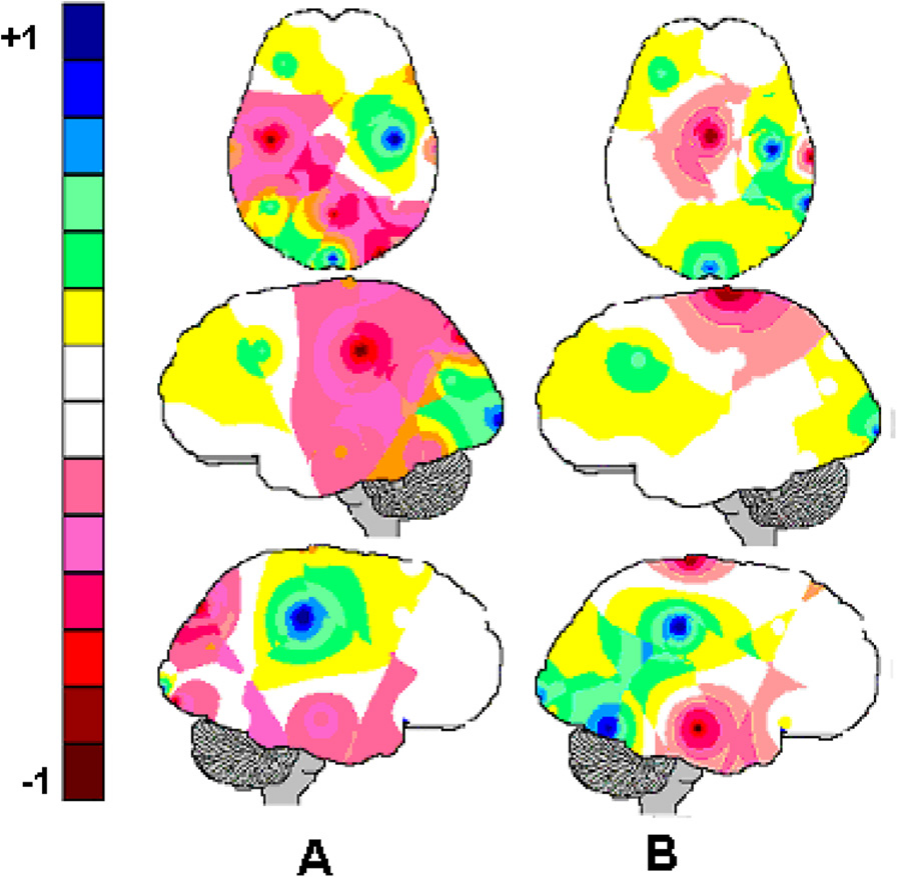
**The brain mappings of the logistic regression between the diagnostic decision, d (dependent variable; wrong = 0, right = 1), and *H*(*e***_***i***_**), which were calculated for each of the electrodes (independent variables).** The normalized $\beta_{i}\bar{H}\left(e_{i}\right)$ values obtained for each electrode were color-coded such that yellow to dark-blue areas were associated with increasingly values of positive *β*_*i*_ and pink to dark-red areas were associated with increasingly values of negative *β*_*i*_. The areas in white were not statically significant and the difference between the mappings in **A** (see Table 3) and B (see Table 4) is the use of expertise as a control variable in **B**.

The logistic brain mapping calculated from the normalized values $\beta_{i}\bar{H}\left(e_{i}\right)$ in Table 3 is shown in Figure 6A and the mapping calculated from Table 4 is shown in Figure 6B.

In the case of Table 3 and Figure 6A, the high values of *H*(*e*_*i*_) calculated for C4, F3, O1, OZ and P3 were associated with correct diagnosing because the values of *β*_*i*_ obtained for these electrodes were positive. In contrast, the high values of *H*(*e*_*i*_) calculated for C3, O2, PZ, T4 and T5 were associated with incorrect diagnosing because the values of *β*_*i*_ obtained for these electrodes were negative. The use of *expertise* as a control variable (Table 4 and Figure 6b) had changed these correlations such that the high values of *H*(*e*_*i*_) calculated for C4, F3, F8, OZ and T6 were associated with correct diagnosing and the high values of *H*(*e*_*i*_) calculated for CZ and T4 were associated with incorrect diagnosing. These two brain mappings were correlated at the level of 0.59 as evaluated by Pearson correlation coefficient and the main differences between the two were observed for the electrodes with negative *β*_*i*_*’*s. In other words, the main differences involved the electrodes for C3, PZ and T5, that exhibited high entropy values *H*(*e*_*i*_) and were associated with incorrect diagnostics, and P3 and O1, which displayed high entropy values *H*(*e*_*i*_) are associated with correct diagnostics.

**Figure 7:**
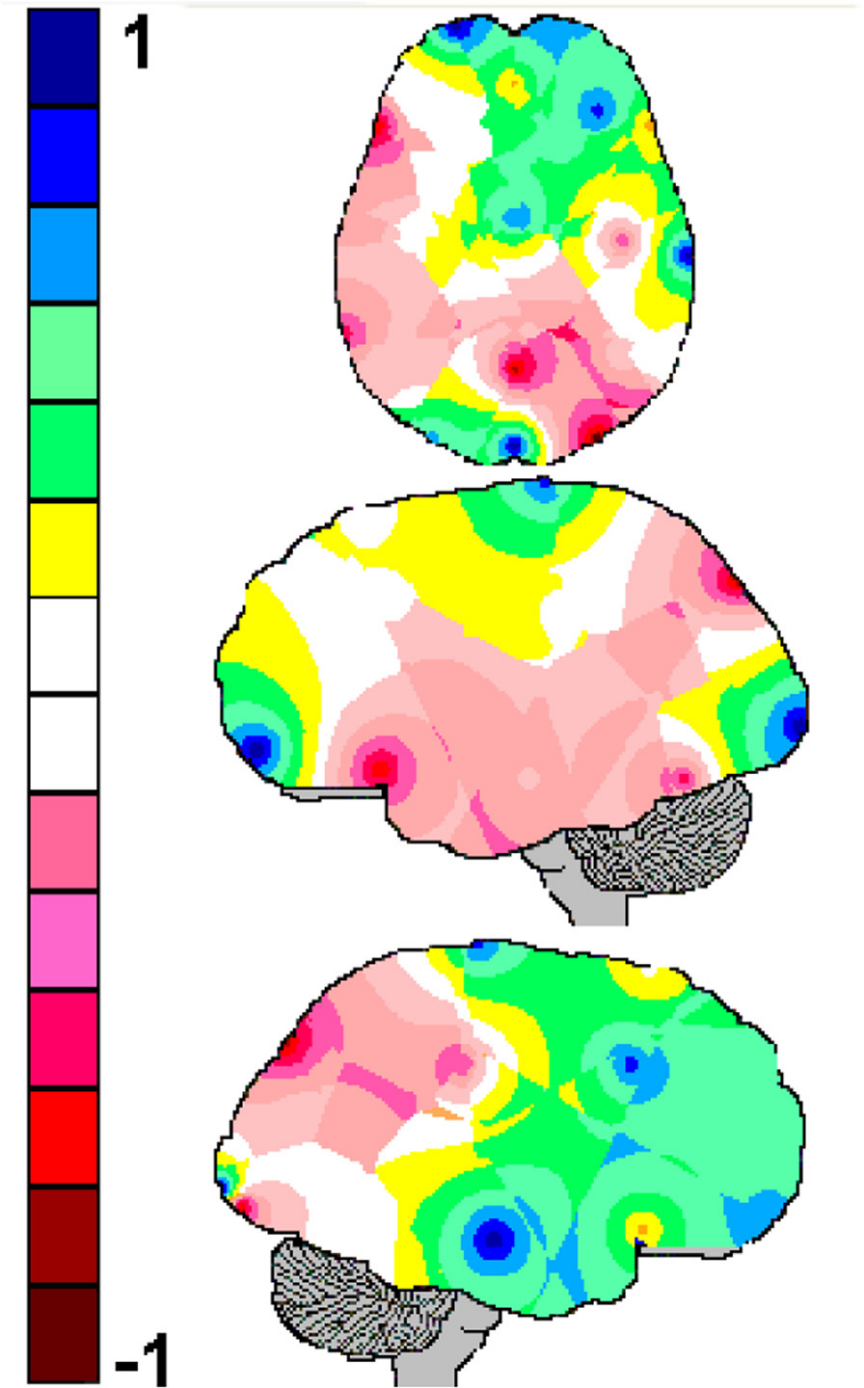
**The brain mappings of the regression between expertise and *H*(*e***_***i***_**) calculated for each of the electrodes (independent variables).** Color-coding is similar to that in Figure 6.

Table 5 shows the results of the logistic regression analysis between the expertise (as measured by the years of practice) and the *H*(*e*_*i*_) calculated for each of the electrodes. This regression explains approximately 34% of the observed data. The regression brain mapping calculated from the normalized values $\bar{\beta_{i}H\left(e_{i}\right)}$ in Table 5 is shown in Figure 7.

**Table 5:** **The logistic regression analysis between *expertise *and *H*(*e***_***i***_**)**

**Dependent variable: expertise**				
**Final loss: 234.44**		**R = 0.58**	**R**^**2**^ **= 0.34**	
				**H-B**
	***β***_***i***_	**Std.Err.**	**p-level**	$\beta_{i}\bar{H}\left(e_{i}\right)$
Intercept	-0.02	0.09	0.00	
C3	-0.06	0.09	0.508	0.50
C4	-0.23	0.09	0.010*	0.46
CZ	0.34	0.09	0.000*	0.86
F3	-0.18	0.08	0.019	0.50
F4	0.38	0.08	0.000*	0.89
F7	-0.41	0.06	0.000*	0.33
F8	-0.10	0.06	0.104	0.50
FP1	0.49	0.06	0.000*	0.97
FP2	0.25	0.06	0.000*	0.80
FZ	-0.09	0.07	0.217	0.50
O1	0.23	0.08	0.006*	0.78
O2	-0.88	0.07	0.000*	0.00
OZ	0.54	0.06	0.000*	1.00
P3	0.09	0.08	0.275	0.50
P4	-0.07	0.08	0.384	0.50
PZ	-0.61	0.08	0.000*	0.19
T3	-0.26	0.06	0.000*	0.44
T4	0.50	0.07	0.000*	0.98
T5	-0.28	0.07	0.000*	0.42
T6	-0.05	0.07	0.449	0.50

The values $\beta_{i}\bar{H}\left(e_{i}\right)$ are the normalized values of the product *β*_*i*_*H*(*e*_*i*_), which were estimated for those inferences according to the Holm-Bonferroni (H-B) method. The statistically significant inferences are marked with asterisks.

The high values of *H*(*e*_*i*_) calculated for CZ, F4, FP1, FP2, O1, OZ and T4 were positively associated with expertise because the values of *β*_*i*_ obtained for these electrodes were positive. In contrast, the high values of *H*(*e*_*i*_) calculated for C4, F7, O2, PZ, T3 and T5 were negatively associated with expertise because the values of *β*_*i*_ obtained for these electrodes were negative.

## Discussion

The difficulty of diagnostic decision-making for the present set of clinical cases was considered to be appropriate for the expertise of the studied veterinary radiologists because they correctly diagnosed 71% of the cases and the total processing time for each case was approximately one minute. In addition, solving the cases involved an acceptable level of cognitive effort because only 31% of the radiologists needed to review the data to reach a final solution.

The Z-score analysis clearly demonstrated that entropy *H*(*e*_*i*_) calculated for all the 20 electrodes was statistically significant because the null-hypothesis was rejected. The *H*(*e*_*i*_) brain maps in Figure 3 showed no differences between the distinct stages (CH, XR and DG) of the clinical reasoning process as demonstrated by the high Pearson correlation coefficient values (see Table 1). Interestingly, the highest values of *H*(*e*_*i*_) were obtained for the frontal electrodes F3, F4, F7, F8, FP1, FP2 and FZ, as well as for the right occipital-parietal electrodes. The lowest values of *H*(*e*_*i*_) were computed for the electrodes C3, C4, CZ and P3 that were the main components of the principal components analysis (PCA) pattern P_2_.

The PCA brain mappings in Figure 4 revealed the existence of 3 patterns of brain activity (P_1_, P_2_ and P_3_) that are very similar for the 3 stages (CH, XR, DG) of the clinical reasoning process, as demonstrated by the high values of their Pearson correlation coefficients (Table 2). Pattern P_1_ consists of all the frontal electrodes; pattern P_2_ is bilateral and consists of the temporal-central electrodes, although T5 is missing in the DG phase. Finally, pattern P_3_ predominantly consists of the occipital-parietal electrodes, although it also includes T6.

Intriguingly, the patterns P_1_ and P_3_ overlap with the electrodes that exhibit the highest mean entropy (Figure 2), whereas pattern P_2_ includes most of the electrodes that exhibits the lowest values of the mean entropy. A possible explanation for these findings is to assume that diagnostic reasoning is supported by neural networks of small-world type [36] in which some areas play the role of hub nodes that are well connected to all the other complementary nodes (areas) recruited in the same processing. These hub nodes are supposed, therefore, to have high values of *H*(*e*_*i*_) in comparison to the complementary nodes.

These results appear to confirm our assumption that brain activity patterns during the CH, XR and DG stages did not differ because it was associated with the assessment and processing of the symptom or sign identification uncertainty, prevalence (or premises), and hypothesis (consequence) plausibility. The Pearson correlation coefficients were very high for all pattern comparisons, which mean that the PCA patterns are similar for all phases of clinical diagnosis. In addition, the loading and score 3-D plotting in Figure 5 did not disclose any remarkable difference between the 3 diagnostic stages, although pattern visual inspection of Figure 4 may provide support to some P_3_ differences between DG stage and CH or XR stages. Therefore, we hypothesize that the three different patterns of brain activity revealed by PCA are to be associated with different tasks that are required by clinical reasoning.

Rocha [4] and Leão and Rocha [7] proposed elsewhere that the clinical decision-making is an analytical process that uses a structured set of rules (e.g., Figure 2) to correctly:

a) weigh the uncertainty of the identification of the sign and symptoms that characterizes a particular disease [4,7];

b) weigh the uncertainty of the prevalence of the above features [4,7]; and

c) combine these features to arrive at the correct diagnosis [4,7].

A word of caution concerning the above hypotheses, however, is necessary because *H*(*e*_*i*_) is a variable that summarizes information about sets of neurons that are activated in the attempt of solving a given task. Therefore, it is quite possible that PCA mappings have disclosed the overall structure of clinical reasoning, as proposed in a to c above without detecting fine-grained details characteristic of each diagnostic phase.

Here, we propose that a key information from the clinical history (e.g., **claudicating of the hind limbs** in Figures 1 and 2) *triggers*[4] a set of structured rules (RG) to be further investigated [6,7,9,11]. Clinical reasoning is the navigation of this RG, taking into consideration the uncertainty of the identification of symptoms and signs and their prevalence. Taking into consideration the literature regarding working memory and executive functions [37-40], we propose that pattern P_1_ (Figure 4) discloses the brain activity that is associated with the recognition of triggers in CH; rehearsal of the corresponding RGs for analysis and the navigation of these reasoning networks according to the uncertainty of the other pieces of information in CH and XR, as well as finally selecting the most plausible diagnosis in DG. Because P_1_ did not differ between the three experimental epochs (CH, XR and DG), this may be interpreted that the same RG was considered during the entire clinical reasoning that was associated with each clinical case. This may be true either if just one RG was triggered for the analysis in the simple cases, or more than one RG were being taken into consideration in the complex cases.

Identification uncertainty assessment is a task associated with the semantic decoding of both verbal information in CH and visual information in XR. In both cases, this semantic decoding involves the recall of information about the symptoms or signals from memory that were learned from previous cases and/or specialized literature [6,7,9,11]. Identification uncertainty is a direct function of how variable a symptom or signal may be and of how clearly it is observed in the case under consideration. In this experiment, the identification uncertainty in CH is mostly dependent on the former and in XR, it is mostly dependent on the latter.

The assessment of the uncertainty of association between a signal or symptom and a given diagnosis, or prevalence, is also a task associated with semantic decoding in CH and XR. It is also dependent on both the information learned from previous experience and literature. In this study, the prevalence in CH and XR is dependent on strength of the association between the signal or symptom and the diagnosis as learned from the literature and/or previous experience. Here, we propose that pattern P_3_ discloses the brain activity associated with the assessment of both uncertainty of identification and prevalence because it involves electrodes that have been reported to be involved with the semantic decoding of both verbal and visual information [41-44]. Because P_3_ does not vary during the experiment, we have to conclude that the same neural circuits are involved in this uncertainty assessment despite its type and type of symptom or signal.

RG navigation is dependent on using uncertainty of identification and prevalence associated with its terminal nodes (symptoms or signals) to calculate the plausibility of the hypothesis encoded by intermediate or root nodes (see Figure 2). In other words, the clinical reasoning or RG navigation is dependent on using the uncertainty of identification and prevalence about the antecedents of a given rule to calculate the plausibility of the hypothesis encoded by its consequence. Rocha [4] proposed that logic operators of type *and*, *or*, *most of all*, *at least one, etc.* are used to calculate the plausibility of each rule consequent taking into account identification uncertainty and prevalence of each rule antecedents. We propose that pattern P_2_ discloses the brain activity of the neural circuits involved with such logical calculations. The results from neuroeconomic studies have involved central-parietal areas with the calculation of the plausibility of an outcome given the uncertainty about its causes [45,46]. Because similar types of logical calculations were required in CH, XR and DG, the pattern P_2_ should be similar for these three experimental epochs as observed in Figure 4.

The logistic regression analysis (Tables 3, 4 and Figure 5) showed that the correlation between diagnosing (**d**) and *H*(*e*_*i*_) is influenced by *expertise*, which increased R^2^, reducing the number and changed some of the electrodes that were significantly correlated with **d**. This is consistent with the literature showing that experts use a smaller and more structured set of rules in comparison to the novice [4,7,9]. The logistic regression analysis controlled by expertise, showed that activity recorded by the electrodes C4, F3, F8, OZ and T6 is positively correlated with the success in making the correct diagnosis. In other words, the clinical reasoning success increased as the amount of information of the electrical activity recorded by these electrodes increased. On the contrary, the increase in the correlation entropy of the electrical activity recorded by the electrodes CZ and T4 increase was associated with a high probability of an unsuccessful clinical reasoning.

The positive correlation between the correct diagnosis and the amount of information provided by C4, F3, F8, OZ and T6 may be explained if we assume that the increase of *H*(*e*_*i*_) at

a) T6 is associated with a correct assessment of total uncertainty;

b) OZ and C4 is associated with a correct calculation of the hypothesis plausibility and

c) F3 and F8 is associated with the selection of the most plausible clinical and XR hypothesis.

The negative correlation between correct diagnosis and the amount of information provided by CZ and T4 can be explained if we assume that the increase of *H*(*e*_*i*_) calculated for these electrodes is related to the analysis of the alternative wrong hypothesis that in some cases were assumed to be the most plausible and induced the volunteer to make a wrong decision.

A comparison of the mappings A and B, in Figure 5, shows that the adjustment of the logistic model by the variable '*expertise*’ mostly reduced the number of electrodes that were correlated with wrong diagnosing. More specifically, the activity recorded by the electrodes C3, P3, PZ, T5 and O2 became less influential in the diagnostic decision-making as the *expertise* progressed. Here, the inference assuming correct diagnosis to be negatively correlated with the amount of information provided by CZ did not reach the Holm-Bonferroni significance criteria (*p* < 0.007), although its calculated *p-level* resulted 0.039. In addition, the regression analysis showed that *expertise* correlates with *H*(*e*_*i*_) (Table 5 and Figure 6) such that a high *H*(*e*_*i*_) at FP1, FP2, F4, CZ, T4, O1 and OZ is associated with long clinical practice and the opposite is true for the electrodes F7, PZ, T3, T5 and O2. In other words, the neural circuits support clinical reasoning to reorganize themselves as the number of years for clinical practice increases. Such reorganization increases the correlation entropy of FP1, FP2, F4, CZ, T4, O1 and OZ and decreases that of PZ, T3, T5 and O2. It may be assumed therefore, that most of the electrodes recording the activity associated with patterns P_1_ and P_3_ increase *H*(*r*_*i*_) with clinical practice whereas the opposite is true for the majority of the electrodes that compose the pattern P_2_ (compare Figures 4 and 6). Such results clearly show that novices and experts use different mental strategies to reason clinical cases because the EEG activity is modulated by the number of years of clinical experience. Although the literature [9,11] on clinical reasoning have already raised the hypothesis that novices and experts use different reasoning strategies, to the best of our knowledge, this is the first study to provide empirical support for this hypotheses.

In a recent study Melo et al. [12] designed a very specific fMRI protocol to analyze pattern recognition in clinical diagnosis and proposed the hypothesis that doctors recognize XR signals through lexical-semantic associations. Here, we show that clinical diagnosis is a much more complex process, which includes pattern recognition (as in XR signal identification) and analogical or analytical reasoning.

## Conclusions

Summarizing the above conclusions, we propose that the volunteers in this study used the information obtained from the clinical history to trigger one or more diagnostic hypotheses and then used these hypotheses to recall X-ray information from memory. They ultimately used this recalled information to make decisions about X-rays in their daily clinical practices. Uncertainty about the identification of both the clinical and XR data and the prevalence of these pieces of information was used to calculate the hypothesis plausibility. In this context, we may conclude the following:

1. PCA analysis was successful in disclosing the different patterns of brain activity associated with hypothesis triggering and handling (pattern P_1_); identification uncertainty and prevalence assessment (pattern P_3_), and hypothesis plausibility calculation (pattern P_2_);

2. Logistic regression analysis was successful in disclosing the brain activity associated with clinical reasoning success, and together with

3. Regression analysis showed that clinical practice reorganizes the neural circuits supporting clinical reasoning.

### Ethical aspects

This study was approved by the Ethics Committee of the School of Medicine of the University of São Paulo.

## Competing interests

The authors declare that they have no competing interests.

## Authors’ contributions

EMand AFda R made contributions to the study design, established the experimental model and drafted the manuscript. LMRand FTR carried out the data collection and analysis of the EEG and NRSO carried out the statistical analysis. All authors read and approved the final manuscript.

## References

[B1] Massad E Ortgea NRS de Barros LC Struchiner CJ Fuzzy logic in action: applications in epidemiology and beyond 2008 Berlin, Heildelberg, New York: Springer Verlag

[B2] Magiameli P West D Rampal R Model selection for medical diagnosis decision support systems Dec Supp Syst 2004 36 3 247 259 10.1016/S0167-9236(02)00143-4

[B3] Pandey B Mishra RB Knowledge and intelligent computing system in medicine Comp Biol Med 2009 39 3 215 230 10.1016/j.compbiomed.2008.12.008 19201398

[B4] Rocha AF Neural Nets: A Theory for Brains and Machine. Lecture Notes in Artificial Intelligence 1992 Heidelberg: Springer-Verlag

[B5] Rocha AF Rocha FT Burattini MN Massad E Neurodynamics of an election Brain Res 2010 1351 198 211 20599820 10.1016/j.brainres.2010.06.046

[B6] Bordage G Connell KJ Chang RW Gecht MR Sinacore JM Assessing the semantic content of clinical case presentations Acad Med 1997 72 S37 S39 10.1097/00001888-199710001-00013 9347733

[B7] Leão BF Rocha AF Proposed methodology for knowledge acquisition – a study on congenital heart-disease diagnosis Meth Info Med 1990 29 1 30 40 2407931

[B8] Dosenbach NUF Fair DA Miezin FM Cohen AL Wenger KK Dosenbach RAT Fox MD Snyder AZ Vincent JL Raichle ME Schlaggar BL Petersen SE Distinct brain networks for adaptive and stable task control in humans Proc Nat Acad Sci 2007 104 11073 11078 10.1073/pnas.0704320104 PMC1904171 17576922

[B9] Mandin H Jones A Woloschuck W Harasym P Helping students learn to think like experts when solving clinical problems Acad Med 1997 72 173 179 10.1097/00001888-199703000-00009 9075420

[B10] Knottnerus JA The evidence base of clinical diagnosis 2002 London: BMJ Books

[B11] Norman G Research in clinical reasoning: past history and current trends Med Educ 2005 39 418 427 10.1111/j.1365-2929.2005.02127.x 15813765

[B12] Melo M Scarpin DJ Amaro E Jr Passos RB Sato JR Friston KJ Price CJ How doctors generate diagnostic hypotheses: a study of radiological diagnosis with functional magnetic resonance imaging PLoS One 2011 6 12 e28752 doi:10.1371/journal.pone.0028752 10.1371/journal.pone.0028752 22194902 PMC3237491

[B13] Bland AR Schaefer A Electrophysiological correlates of decision making under varying levels of uncertainty Brain Res 2011 1417 55 66 21911213 10.1016/j.brainres.2011.08.031

[B14] Cohen MX Elger CE Ranganat C Reward expectation modulates feedback-related negativity and EEG spectra Neuro Image 2007 35 968 978 17257860 10.1016/j.neuroimage.2006.11.056 PMC1868547

[B15] Davis CE Hauf JD Wu DQ Everhart DE Brain function with complex decision making using electroencephalography International J Psycho physiol 2011 79 175 183 10.1016/j.ijpsycho.2010.10.004 20955738

[B16] Selimbeyoglu A Keskin-Ergen Y Demiralp T What if you are not sure? Electroencephalographic correlates of subjective confidence level about a decision Clin Neuro physiol 2012 123 1158 1167 10.1016/j.clinph.2011.10.037 22119799

[B17] Karch S Feuerecker R Leicht G Meindl T Hantschk I Kirsch V Ertl M Lutz J Pogarell O Mulert C Separating distinct aspects of the voluntary selection between response alternatives: N2- and P3-related BOLD responses Neuroimage 2010 51 356 364 10.1016/j.neuroimage.2010.02.028 20171291

[B18] Polezzi D Sartori G Rumiati R Vidotto G Daum I Brain correlates of risky decision making Neuroimage 2010 49 1886 1894 10.1016/j.neuroimage.2009.08.068 19761850

[B19] Tsovara A Murray MM Bourdaud N Chavarriaga R Millán JR De Luci M The timing of exploratory decision-making revealed by single-trial topographic EEG analyses Neuroimage 2012 60 1959 1969 10.1016/j.neuroimage.2012.01.136 22342874

[B20] Cohen MX Ridderinkhof KR Haupt S Elger CE Fell J Medial frontal cortex and response conflict: evidence from human intracranial EEG and medial frontal cortex lesion Brain Res 2008 1238 31 127 142 18760262 10.1016/j.brainres.2008.07.114

[B21] Esposito F Mulert C Goebel R Combined distributed source and single-trial EEG–fMRI modeling: application to effortful decision making processes Neuroimage 2009 47 112 121 10.1016/j.neuroimage.2009.03.074 19361566

[B22] Jacobs J Hwang G Curran T Kahana MJ EEG oscillations and recognition memory: theta correlates of memory retrieval and decision making Neuroimage 2006 32 978 987 10.1016/j.neuroimage.2006.02.018 16843012

[B23] Lindsen JP Jones R Shimojo S Bhattacharya J Neural components underlying subjective preferential decision making Neuroimage 2010 50 1626 1632 10.1016/j.neuroimage.2010.01.079 20116436

[B24] Dien J Spencer KM Donchin E Localization of the event-related potential novelty response as defined by principal components analysis Cogn Brain Res 2003 17 637 650 10.1016/S0926-6410(03)00188-5 14561451

[B25] Maguire MJ Brier MR Ferree TC EEG theta and alpha responses reveal qualitative differences in processing taxonomic versus thematic semantic relationships Brain Lang 2010 114 16 25 10.1016/j.bandl.2010.03.005 20403632

[B26] Mouraux A Iannetti GD Across-trial averaging of event-related EEG responses and beyond Magn Reson Imag 2008 26 1041 1054 10.1016/j.mri.2008.01.011 18479877

[B27] Rocha AF Massad E Pereira A Jr The brain: from fuzzy arithmetic to quantum computing 2004 Heildelberg: Springer Verlag

[B28] Rocha FT Rocha AF Massad E Menezes RX Brain mappings of the arithmetic processing in children and adults Cogn Brain Res 2005 22 359 372 10.1016/j.cogbrainres.2004.09.008 15722207

[B29] Foz FBA Luchini S Palmeri AF Rocha EC Rodela AG Rondó M Cardoso PB Ramazzini C Leite C Language plasticity revealed by EEG mapping Pediat Neurol 2001 26 106 115 11897474 10.1016/s0887-8994(01)00368-x

[B30] Rocha AF Rocha FT Massad E The brain as distributed intelligent processing systems: an EEG study PLoS One 2011 6 3 e17355 doi:10.1371/journal.pone.0017355 10.1371/journal.pone.0017355 21423657 PMC3057967

[B31] Hauk O Davis MH Ford M Pulvermüller F Marslen-Wilson WD The time course of visual word recognition as revealed by linear regression analysis of ERP data Neuroimage 2006 30 1383 1400 10.1016/j.neuroimage.2005.11.048 16460964

[B32] Delarme A Makeig S EEGLAB: an open source toolbox for analysis of single-trial EEG dynamics including independent component analysis J Neurosci Meth 2004 134 9 21 10.1016/j.jneumeth.2003.10.009 15102499

[B33] Olofsson JK Nordin S Sequeira H Polich J Affective picture processing: an integrative review of ERP findings Biolog Psychol 2008 77 247 265 10.1016/j.biopsycho.2007.11.006 PMC2443061 18164800

[B34] Polezzi D Daum I Rubaltelli E Lotto L Civai C Sartori G Rumiati R Mentalizing in economic decision-making Behav Brain Res 2008 190 218 223 10.1016/j.bbr.2008.03.003 18406476

[B35] Heldmann M Russeler J Munte TF Event-related potentials in a decision-making task with delayed and immediate reward conditions J Psycho physiol 2005 19 270 274 10.1027/0269-8803.19.4.270

[B36] Iturria-Medina Y Sotero RC Canales-Rodríguez EJ Alemán-Gómez Y Melie-Garcíaa L Studying the human brain anatomical network via diffusion-weighted MRI and Graph Theory Neuroimage 2008 40 1064 1076 10.1016/j.neuroimage.2007.10.060 18272400

[B37] Andrés P Frontal cortex as the central executive of working memory: time to revise Our view Cortex 2003 39 871 895 10.1016/S0010-9452(08)70868-2 14584557

[B38] Baddeley A The episodic buffer: a new component of working memory? Trends Cogn Sci 2000 4 417 423 10.1016/S1364-6613(00)01538-2 11058819

[B39] Michels L Moazami-Goudarzi M Jeanmonod D Sarnthein J EEG alpha distinguishes between cuneal and precuneal activation in working memory Neuroimage 2008 40 1296 1310 10.1016/j.neuroimage.2007.12.048 18272404

[B40] Wu X Chen X Li Z Han S Zhang D Binding of verbal and spatial information in human working memory involves large-scale neural synchronization at theta frequency Neuroimage 2007 35 1654 1662 10.1016/j.neuroimage.2007.02.011 17379539

[B41] Bechtereva NP Korotkov AD Pakhomov SV Roudas MS Starchenko MG Medvedev SV PET study of brain maintenance of verbal creative activity *Int* J Psycho physiol 2004 53 11 20 10.1016/j.ijpsycho.2004.01.001 15172131

[B42] Cabeza R Nyberg L Imaging cognition: an empirical review of PET studies with normal subjects J Cogn Neurosci 1997 9 1 26 10.1162/jocn.1997.9.1.1 23968177

[B43] Martín-Loeches M Casado P Hernández-Tamames JA Álvarez-Linera J Brain activation in discourse comprehension: a 3t fMRI study Neuroimage 2008 41 614 622 10.1016/j.neuroimage.2008.02.047 18394923

[B44] Pulvermüller F Shtyrov Y Hauk O Understanding in an instant: Neurophysiological evidence for mechanistic language circuits in the brain Brain Lang 2009 110 81 94 10.1016/j.bandl.2008.12.001 19664815 PMC2734884

[B45] Siebörger FT Ferstl EC Yves von Cramon D Making sense of nonsense: an fMRI study of task induced inference processes during discourse comprehension Brain Res 2007 1166 77 91 17655831 10.1016/j.brainres.2007.05.079

[B46] Lau B Glimcher PW Value representations in the primate striatum during matching behavior Neuron 2008 58 451 463 10.1016/j.neuron.2008.02.021 18466754 PMC2427158

